# Influence of COVID-19 Pandemic on Psychological Status: An Elaborate Review

**DOI:** 10.7759/cureus.29820

**Published:** 2022-10-01

**Authors:** Sakshi Kamble, Abhishek Joshi, Ranjit Kamble, Smita Kumari

**Affiliations:** 1 Psychiatry, Jawaharlal Nehru Medical College, Datta Meghe Institute of Medical Sciences, Wardha, IND; 2 Community Medicine, Jawaharlal Nehru Medical College, Datta Meghe Institute of Medical Sciences, Wardha, IND; 3 Department of Orthodontics, Sharad Pawar Dental College, Datta Meghe Institute of Medical Sciences, Wardha, IND

**Keywords:** corona virus, depression, mental health, social distancing, covid-19

## Abstract

The 21^st^ century has seen a spike in virus outbreaks. The Coronavirus infection in 2019, originating from Wuhan Province, China, spread across the world and was declared a pandemic. It led to the imposition of lockdowns in different parts of the world, as lockdowns were an effective and essential way to break down the cycle of infection. Physical distancing was the most significant measure to break the infection cycle. The Coronavirus infection outbreak has seen a concurrent spike in mental health risks worldwide. Mental health is considered to be one of the important components of overall health issues. A good mental health state is when people are stable with the environment and should not be effortlessly upset. They should know their requirements, difficulties, and goals properly; if they face problems, a trial should be done to solve them logically to cope with stress and anxiety. COVID-19 has set off a wide variation of mental issues, for example, anxiety, panic disorder, and depression. Most of the studies have reported negative impacts, including anger, stress indications, and confusion. Healthcare providers who have worked around the look after patients had also found a need for emphasis on mental health. Mental health awareness among the general population has gained importance since the high spike rise of mental health issues. This has garnered popularity in the general population and clinicians to pay due attention to mental health. Awareness and measures to keep mental health issues at bay need to be emphasized and undertaken. This organized review targets to highlight the psychological impact on the general population and associated risk factors that are components of aggravation.

## Introduction and background

Atypical variants of pneumonia were reported in Wuhan, Central China, in December 2019; recognized by the WHO as "Coronavirus disease 2019 (COVID-19)". The virus "SARS-CoV-2" had a unique strain that shared 79% genetic likeness with "SARS-CoV" from the 2003 SARS outburst. WHO had alarmed an emergency in March 2020 regarding the COVID-19 pandemic and psychological concerns, including emotional state, substance abuse, and suicidal tendencies. Tension and depression tendency had a marked prevalence of 41%, according to a survey in January 2021. The suicidal tendency has found a rise that marks the worsening of the situation across the globe. In early 2020, drug abuse and death due to overconsumption of drugs were witnessed. As humans are social animals; the pandemic did disturb their state of mind. The world came to know the dark side of being devoid of human touch and being constrained in closed spaces. This affected the sleep cycle, and sleep is one of the body's natural healers. Post a good sleep, a person loses attention towards unpleasant events, thereby being renewed with energy and zeal. When this is hampered, it leads to suicidal tendencies. In India, lockdown marked a rise in disturbing mental states like stress, anxiousness, insomnia, etc. The impact on the economy was enormous as a huge population of the country relies on their daily income. The depleted income led to hunger, insecurity, and loss of home. Loan defaulters increased, which shook confidence in a person's credibility in the public sphere impacting mental health. Unemployment and poverty saw a rise across the globe. Child abuse also rose as they are an easy target to vent anger. Parents found it hard to manage and engage kids at home. Children lost healthy exercise routines, and were devoid of sports, which not only affected their physical but also mental well-being. Awareness drives need to be undertaken to identify disturbed mental states and measures to prevent their occurrence and how to seek expert care to get cured.

## Review

Bilal Javed et al. [1] studied the impact of the COVID-19 pandemic on mental health. When all workforce of the medical and allied fraternity was contributing to epidemiology, clinical structures, patterns of transmission, and managing the outbreak, somehow no light was put on the impact of a pandemic on mental health. The concept of quarantine was the only solution the human race had, but it was one of the worst punishments. A person is confined to a small space, and inactivity affected mental well-being massively. Childhood is the best phase of a human lifetime, where social mannerisms develop and societal bonding comes into a role. Due to the pandemic, children were devoid of it, thereby affecting their mental well-being. The quarantine and isolation period not only affected the mental health of persons who had exposure to the virus but also their families because of a lack of face-to-face communication. Understanding the impact of the "COVID-19" pandemic on the mental health of numerous populations is as significant as understanding its clinical features, communication designs, and management.

Esther Crawley et al. [2] found that parental mistrust leads to a decline in vaccination and the manifestation of aggravated conditions in children. “COVID-19” emergency has caused deaths across all age groups. The shutting down of schools did not substantially decrease the death rate, but it had a greater impact on education quality, which could have been rather better when knowledge would be imparted in school and wisdom acquired by being in the social sphere. This had the adverse effect of the feeling of isolation. European countries in greater numbers had declared a shutdown of schools to control the spread of the pandemic. Not only in Europe but children worldwide got deprived of the greatest privilege of being schooled with their freedom. The worst sufferer’s being economically less privileged communities who had limited access to the internet. This further aggravated their mental well-being by not working toward a better life. The limited healthcare facility towards milder forms of ailment had further caused their progression to severe ones as healthcare services were focusing on COVID-19 cases outburst and very severe ailment which were in the category of highest emergency. This would thus not only affect physical but also mental well-being.

Baker and Clark et al. [3] concluded that when healthcare providers have to decide to deliver services with a limited workforce, the older group gets less attention as the workforce has to choose the ones in more need of care. The older group is thus more prone to isolation, affecting their mental health. Healthcare and allied providers had found that even in the pre-COVID-19 era, availing of healthcare services was not just for health issues but also to find a place to go to communicate with other people face-to-face. In this era, everyone is busy, and finding time to sit with the elderly is difficult. Thus, the COVID-19 scenario adversely affected the elderly by increasing their loneliness, boredom, and isolation. The UK government had imposed stricter guidelines of physical distancing and isolation for protection or prevention of their older citizens with heavy hearts but considering the need of the hour.

Zhao et al. [4] scrutinized social distancing and its impact on mental health. The Hong Kong COVID-19 Health Material Survey April 2020, through telephonic interviews and online surveys, collected data about stress, anxiety, and depressing symptoms and analyzed the subjects. The preventive measures to handle the pandemic were "isolation, quarantine, contact tracing, and physical distancing". Most defendants had remained indoors for at least four of the previous seven days. Home isolation had been found to produce fewer stress levels when compared to hospital isolation but inactivity during that period had its impact on the mental well-being of the patient. Mental well-being was affected by the loneliness that aggravated the stress, anxiety, and mild depression produced by the disease. As in most affected states, the caretakers of patients provide care being near to the patients, and they provide great mental support.

Saladino et al. [5] studied the evaluation of the psychological and social effects of the pandemic on the population. The severity of mental well-being due to loneliness can be made less severe by means of telephonic consultation and counselling, thereby preventing progression of the agony. COVID-19 has brought in a fear of exposure to virus with every human touch. It has brought with it a vicious cycle of depression and slower rate of healing as mental support in person has greatest impact on healing from depression. The world has witnessed many viral outbreaks over the years but COVID-19 severely affected the human race amongst all. This has increased the rate of suicidal tendency, which is the last stage of depressive condition or worst manifestation. Anxiety was common among children and young adults. The guardians of children found that concentration was declining in children. Also loneliness was felt by children at home, restlessness and irritability had a rise in children.

Healthcare providers were working with less workforce and overtime to fill the gap, which caused their mental health to detritions. The exposure of patients and transmission to family was also stressful to healthcare workers. The author concluded that psychologists and psychotherapists should be trained in providing online services. The general population needs to be made aware of the new services and should be encouraged to avail services before mental health deteriorates.

L Masse et al. [6] conducted a study to attain a relationship between the financial situation of a family and associated psychological factors that came along to combat the hard times of the pandemic. Due to the lockdown, there were challenging situations economically. There was restricted mobility, and everything associated with social life had to come to a halt. In this survey, it was found that children who belonged to a better financial background were somewhat less prone to developing mental and physical ailments as financial well-being allowed these children and their families to connect to other people via telephone and were able to afford any kind of help which was needed easily. A finding of higher levels of financial well-being was associated with better mental and physical benefits in children during the COVID-19 pandemic. 

Beatrice Ravizza et al. [7] surveyed with the help of pediatricians and child psychiatrists to reflect on the effects of the pandemic on children and their families. It was concluded that with help of proper information and building strong resilience could help deal with this infection and the stigma associated with it in a better way.

Rachele Mariani et al. [8] studied the trauma that came along with the pandemic and the different ways with which humans adapted to the changes. The results of the study showed that the strict lockdown rules affected the emotional capacity of individuals. People were more prone to land into sadness and depression. The pandemic has negatively affected the ability to interpret a situation and cope with it in day-to-day life.

Panchal et al. [9] studied and correlate the COVID-19 pandemic and the economic slowdown and showed that it had a negative impact on people's state of mind across the globe. This led to people finding solace in substance use. The disturbed sleeping cycle and diet and use of alcohol and addictive substance abuse has worsened the scenario. The shutting down of colleges and fall of income in the family has had its severe blow on mental health of adults. There were manifestations of severe symptoms in adults, like stress and low zeal in everything. Substance abuse and suicide tendency has spiked in the COVID-19 era. Handling kids at home without much aids has impacted the well-being of mothers in particular and guardians of children also. The healthcare workers were worst hit in terms of working at the zenith of their capabilities. The fear for their lives and their loved ones was also a worry in their lives which multiplied their stress level because of overworking at the tip of their toes.

Galea et al. [10] stated that there were associated increases in post-trauma stress disorder (PTSD) and psychological distress in patients and caregivers. In the context of the COVID-19 pandemic, the psychological aspect of COVID-19 times has led to venting of frustration on children. It has appeared that substance abuse and domestic violence with school shutdown has closed all space for children to find solace. Children felt left out. The author suggested that social media needs to be directed towards encouraging groups to seek mental health counselling. Counselling and follow-up when domestic violence against children is reported should have a proper and organized medium for solving the scenario.

Giuntella et al. [11], in their study on lifestyle and mental health disruptions during COVID-19, showed that physical activity was hampered, sleep cycle and daily routine was also affected. Physical activity was measured using the number of steps travelled and span of sleep of the subjects. Though physical activity declined, the sleep span increased. The time spent on various social networking site and smartphone usage increased to a greater amount. Lifestyle standards in terms of healthy routine which was devoid of exercise and productive activities further caused decline in mental well-being there by leading to rise in depression. The well-being of a person not only lies in physical health, but the stability or instability in maintenance is greatly governed by mental health. Travis C M Ting et al. [12] also concluded through their study that this pandemic had taken a psychological and emotional toll on those already having mental ailment.

Kevin Kendrick et al. [13] tried to focus on the different responses of different topographical areas as a result of various practices related to the pandemic. There was a great variation in response, mainly affected by false information, scarcity of completing demands, limited resources, etc., which thereby led to a different kind of response worldwide. Some regions showed a higher incidence of suicidal tendencies along with other psychological ailments. Thereby it was concluded that all health care workers should assess the geographical conditions and accordingly deal with patients helping them come out of this pandemic.

Venkatesh and Edirappuli et al. [14 ] reported that depression and anxiety were found as a result of isolation and quarantine programs during this pandemic. Similar effects were observed due to confinement from their loved ones, being devoid of their liberties, and feeling of lack of purpose due to differentiated routine and livelihood. This leads to low mood, boredom, frustration, and potential depressive disorders. Anxiety arises due to fear of containment and improper clarity regarding guidelines of physical distancing. Handling these mental health problems needs a cumulative effort from policymakers, the public, and healthcare professionals.

Melo and Soares et al. [15] concluded that the COVID-19 pandemic has led to harsh economic losses, decreased physical interactions, and major psychological issues. In a survey on the pandemic based in China, high levels of depressive and anxiety disorders were observed. An American study researched more than 10 million Google searches and found that there were major changes in search patterns regarding mental health after stay-at-home curbs. Searches regarding sleep disturbances, suicidal ideation, anxiety, and negative thoughts increased drastically. The isolation programs caused major negative impacts on psychiatric health within a small time of implementation of the policy, especially in people with low pay grades or temporary jobs. Humans irrespective of their country of residency or culture, and isolation for a long period may lead to psychological distress. The burden on the economy due to the pandemic, along with many jobs lost, aggravated poverty, and inequality, may add to these feelings. Steps like online support on this topic and telephonic communications between friends and family may help relieve these feelings.

Manivannan M et al. [16] through their study, concluded that due to such long times spent at home due to lockdown, there has been an increased risk of developing hysteria and paranoia amongst the population, which has somehow further led to a rise in the number of accidental hurts at home to the suicidal tendency in various age groups, especially in the young individuals suggestive of how deeply COVID-19 pandemic has affected the mental health.

Golechha et al. [17] in his study noted that the COVID-19 pandemic is leading to mental health problems like insomnia, denial, anger, fear, anxiety, stress, depression, and even suicidal idea and attempts worldwide, which led to many chronic disorders. It has also led to alcoholism, increase in gender-based violence, mass unemployment, hunger, homelessness, and loan defaults, with increase in overall poverty. Government of India has developed several measures to counter the mental health problems. The Ministry of Health and Family Welfare has formed helplines for mental health. The Rashtriya Kishor Swasthya Karyakram (National Adolescent Health Programme) played a major role in this change and helped the adolescent age group against mental health troubles that may occur post pandemic.

Browning et al. [18] correlated the effects of COVID-19 on student's mental health. According to the study, the following group of pupils had a greater effect on mental health: women, people in fair or poor health, people with below-average family income, people who knew someone who had COVID-19, people in their 18^th^ to 24^th^ year, and people who spend eight or more hours a day in front of a screen. More or less protected from the negative mental impacts were students with above-average socioeconomic status, who spent at least two hours outside, or who spent less than eight hours on electronic devices. Student isolation, stress, a worsening lack of motivation, and anxiety were the most prevalent changes observed. The symptoms of boredom, imprisonment, despondency, exhaustion, guilt, and sleepiness were also concerning. But a relatively small number of students also showed beneficial effects of the pandemic, such as productivity, optimism, empathy, and adaptation.

Dubey et al. [19], in their survey, found that people developed a feeling of loss of control over situations and started feeling left out and cornered. This was a direct effect of mass quarantine and isolation. People started panicking due to the lockdown, causing fear, anguish, and stress. This feeling of being left out can further aggravate if families are forced to divide for breaking the chain of transmission, the uncertainty of further progression of the disease, lack of adequate supply of essential needs like medical facilities and food, financial crisis due to loss of jobs and daily earnings and the fear of contracting the disease has all the more affected the mental health of individuals. Also, physical distancing and self-isolation created a direct effect on mental health, making people irritable, confused, frustrated, denied of things, lost sleep, depressed, and so much, so suicidal tendencies also developed in many people. With all these things came mandatory unavoidable stress, apprehension, and anxiety allied with poorly recognized transmittable ailment outbursts which can be substantial among higher-risk groups, such as healthcare providers and other frontline professionals, such as police officers, and members of the armed services, among others.

Exposure to this pandemic in hospitals, illness, and death of near and dear ones has created a thought of danger in the minds of people and made a toxic impact on the overall health of everyone. Along with the psychological stress faced by medicos because of patient care, there is additional stress due to lack of resources like personal protective equipment (PPE) and lack of adequate training to control such a pandemic, creating a feeling of self-worthlessness. Also, healthcare providers residing in rented apartment were asked to leave their apartments due to fear of contracting the disease. Many doctors were beaten by the patient’s relatives; if unfortunately, the patient could not be saved, creating a behavioral and functional impairment, thereby leading them to emotional collapse.

Migrant workers live in areas where health and hygiene are already deprived. They are already at a higher risk of contracting such infectious diseases. Due to lack of space, education, and financial loss, they were very much prone to depression which may further worsen due to rules imposed by the government in order to curb the spread of infection.

Mentally challenged people are at an increased risk of incurring both bodily and mental harm due to their lack of awareness of the gravity of the situation, misunderstanding of risk parameters, and lack of hygiene can all make such mentally ill patients furthermore prone to contract this disease in its most lethal form. Also, lack of care for these patients can furthermore create a problem in their general health. There can be a recurrence of any psychiatric episode which was partially treated along with the deterioration of existing signs and symptoms due to the inability to avail of a basic healthcare facility.

To combat such tough times and to emerge victorious basic critical organizations for the delivery of psychological healthcare should be made available both at the personal and community level. Websites and various pages should be created along with toll-free numbers to aid all the problems at the community level and to help them cope with all the stigma of mental health. Online platforms can be aptly used to educate people about the severity and outcome of the disease and to inform them about when and how medical help should be availed.

Crawley E et al. [20] conclude that in the U.K., caregivers were increasingly concerned about the anxiety of parents about visiting the hospitals leading to a decrease in vaccination rates which later precipitated severe illness in normal and vulnerable children. He also emphasized the need for program to minimize this collateral damage in children.

Giiner et al. [21] made a survey on a group of pregnant females in a hospital in Italy. They studied the impact of COVID-19 to improve well-being by putting light on this topic for healthcare workers. The study concluded that there are multifaceted effects of this pandemic on pregnant women. They faced many negative thoughts, mostly concerned with fright. To tackle these concerns, healthcare workers should be acquainted with means to deal with the needs of these women, both physical and mental. OzlemGuner et al. [21] also conducted a similar study that included telephonic interviews of pregnant ladies.

AgataBuo et al. [22] also studied the impact of COVID-19 on pregnant females during their antepartum period. The result included that these women faced higher levels of anxiety, and there was a fear of what will happen next amongst these individuals. To cater to these problems there was an increased need to tactfully handle the distress which came along with the pandemic considering all the psychological sides of their story.

AyiVandiKwaghe et al. [23] conducted an elaborate interview with healthcare workers working nearby COVID-19 patients. The survey explored the various difficulties that arose to help and treat the affected population. The major problem entitled with the job was the stigma that came along, which further influenced the quality of work contributed by these diligent workers. Eftekhar, ArdebiliM, et al., [24] through their study, found out that 80.95% of healthcare workers believed that they would consider themselves guilty if anything tragic happens to their family members. Eight healthcare workers who were interviewed had already lost one or more members due to the pandemic and felt a lot of remorse about the entire situation.

Brittney Riedel et al. [25] also, in their study, concluded that early steps should be undertaken to prevent the mental well-being of these healthcare workers as if not treated, it can lead to severe psychological ailment, agony, and in worst cases even death. Pitchot et al. [26] also through a study, emphasized the need to be careful and to plot a way to avert mental problems in susceptible doctors and nurses to decrease the turmoil they had to encounter due to the unexpected and unexplored threats of this pandemic. AnaelleCaillet et al. [27] conducted a study during both the first and second waves of a pandemic. They studied the psychological disorders encountered by ICU caregivers during the pandemic. Their results concluded that there was comparatively less occurrence of psychological problems amongst healthcare workers during the second phase entirely due to the acquired familiarity with the virus and its treatment along with the experience to deal with it.

Berancki et al., [28] in their study on healthcare professionals' attitude, beliefs, and behavior in COVID-19 and dynamics of the healthcare pathway, concluded that the impact of COVID-19 was seen at various levels of healthcare, including screening, diagnosis, treatment, etc. The gaps in care will act as a catalyst for positive change in better healthcare for all.

Nisticò V et al. [29] conducted a survey on a group of patients with eating disorders to evaluate the severity of symptoms like depression, anxiety, and other psychological conditions as compared to healthy individuals during the lockdown. The study concluded that there was an increased prevalence of psychiatric conditions amongst patients with eating disorders in contrast to healthy controls. 

Everly GS et al. [30], in his study, correlated the phases of psychological response to disaster, pre impact of COVID-19, which are often characterized by chaos and uncertainty. As a result, public health disaster planning and response represent formidable challenges. Although disasters can result from a wide array of hazards, regardless of the agent at work, they may follow a rather predictable trajectory of psychological phases. A heuristic of those phases can provide an opportunity for a more organized disaster mental health response and more efficient utilization of scarce resources.

Stufano A et al. [31] conducted a study to evaluate the well-being of employees at the university. The domains under which well-being was evaluated were anxiety, depression, self-control, general health, etc. The results obtained were suggestive of decreased incidence of psychological symptoms during second wave as compared to first phase. It also showed that age was inversely proportional to mental health issues. All these negative symptoms were as a result of deprived interaction amongst employees as compared to times earlier to the pandemic.

Tousignant OH et al. [32] evaluated how the psychological state of a person affected how well they slept during the COVID-19 epidemic in the United States. Results showed that dealing with the COVID-19 pandemic had an impact on psychological health and decreased sleep quality. Therefore, therapies meant to enhance sleep quality should concentrate on lowering concern and boosting resilience-based coping mechanisms. By contextualizing how the model might be utilized for assessments and responses during broad crises and illustrating correlations between crucial factors, this work contributes both practically and theoretically to the field.

Kerr ML et al. [33] did a study on how parents perceived the COVID-19 crisis and how it affected their burnout, their children's conduct, and their income. Parental burnout was more prevalent, children's stress behaviors were increasing, and good child behavior was declining. For families with lesser incomes, there was a higher association between psychological effects and children's stress behaviors.

Tull MT et al. [34] evaluated the impact of COVID-19 and orders to stay at home on psychological outcomes (depression, health anxiety, financial worry, social support, and loneliness) in a U.S. community adult sample across the nation. Orders to stay at home were associated with increased degrees of health concern, financial worry, and loneliness. Additionally, financial concern, social support, and health anxiety were all favorably connected with perceived COVID-19 effects on daily living, while loneliness was adversely correlated.

Li J, Su Q et al. [35] examined the impact of unexpected, life-threatening sickness on healthcare and the psychological pressure that the illness places on nurses. The COVID-19 epidemic has stronger effects on nurses' somatic symptoms and psychological well-being. Compared to non-frontline nurses, frontline nurses had a considerably higher prevalence of moderate and severe PTSD. In addition, they lose weight and experience acute sleeplessness compared to non-frontline nurses.

Guo AA et al. [36] evaluated the effects of COVID-19 on stress and anxiety in undergraduate medical students. First- through fourth-year medical students in the United States were invited to take part in a national online survey that was sent out via email chains between June and August 2020. 4-point demographic data 7-point Perceived Stress Scale for measuring stress. The Generalized Anxiety Disorder Scale was used to quantify anxiety as well as the effects of COVID-19-related social, physical, and academic stressors. According to the study, second- through fourth-year students experienced the most stress. Significantly greater stress and anxiety levels, as well as a higher percentage of stress that could be linked to the COVID-19 were seen in students who already had mental health concerns. The biggest stressor for third-year students was the delay of the standardized tests. The biggest stressor for fourth-year students was the impact on rotations and residencies. Understanding how COVID-19 has affected kid's anxiety and stress.

Wright K et al. [37] evaluated the psychiatric effects of COVID-19. He found that risk populations include children, parents, pregnant women, the elderly, and patients with pre-existing mental health disorders. It increases suicidal tendencies. Medical healthcare workers have increased depression, anxiety, distress, and decreased sleep quality, with female nurses reporting the most symptoms. COVID-19 patients have a high prevalence of post-traumatic stress disorder (PTSD), depression, and poor quality of life. Depression, guilt, and grief are of long-term concern. 

Sarangi A et al. [38] examined the worsening outcomes of the COVID-19 lockdowns on alcoholic patients. He concluded that COVID-19 caused severe anxiety, fear, and psychosocial distress worldwide. Implementation of quarantines by nationwide lockdown had a major effect on mental health disorders, such as depression, anxiety, and especially alcohol use disorder (AUD). The psychosocial consequences of lockdown are isolation, freedom loss, and separation from loved ones.

Many studies were done to improve the mental and physical health of people during COVID-19. Kulkarni MS et al. [39] concluded that yoga has beneficial effects on mental health. Yoga is an intervention in young and adult people for the treatment of both acute and chronic health problems, and also yoga is most effective in reducing symptoms of depression, anxiety, and pain, according to McCall MC et al. [40]. Breedvelt JJF et al. [41] reported that positive effects were observed with mindfulness, yoga, or meditation-based interventions on symptoms of depression, anxiety, and stress.

**Table 1:** Psychological impact of COVID-19 in individuals

Study/year	Individual	Observation/outcome
Esther(2020)[2] , L Masse(2021)[6] , Galea(2020)[10] , Melo(2020)[15] , Browning(2021)[18] , Dubey(2020)[19] , Kerr ML(2021)[33], Wright K(2020)[37]	Parent	Economic problem, mental health
Saladino(2020)[5] ,Melo(2020)[15] , Kevin Kendrick(2021)[13] , Manivannan M(2021)[16] , Dubey(2020)[19], Wright K(2020)[37]		Suicidal tendency
Rachele(2021)[8] , Ting(2021)[12] , Venkatesh(2020)[14] , Golechha(2020)[17] , Dubey(2020)[19] , Crawley (2020)[20] , Nisticò V(2021)[29] , Kerr ML(2021)[33], Sarangi A(2021)[38]		Depression, sadness, affects emotional capacity
Panchal N(2021)[9] , Golechha(2020)[17] , Tousignant OH(2021)[32] , Wright K(2020)[37], Sarangi A(2021)[38]		Disturb sleep cycle, diet, alcohol and addictive abuse
Giuntella(2021)[11]		Physical activity hampered; daily routine affected
Bilal Javed(2020)[1], Wright K(2020)[37]	Children	Affects mental well-being
Esther(2020)[2] , Browning(2021)[18] , Stufano A(2022)[31] , Kerr ML(2021)[33] , Guo AA(2021)[36]		Education quality, feeling of isolation
Saladino(2020)[5] , Ravizza(2021)[7] , Guo AA(2021)[36]		Anxiety, loneliness, irritability, restlessness
Saladino(2020)[5] , Ayi Vandi Kwaghe(2021)[23] , Brittney Riedel(2021)[25] , Pitchot(2020)[26] , Anaelle Caillet(2021)[27], Li J(2021)[35], Wright K(2020)[37]	Doctor/ health care	Affects mental health
Panchal(2021)[9], Eftekhar(2021)[24], Everly GS(2021)[30]		Fear of their life and their family, stress
Baker(2020)[3]	Elder	Loneliness, boredom, isolation, affect mental health
Zhao(2020)[4]		Affect mental health, stress, aggressive, anxiety, mild depression
Ozlem Guner(2022)[21]	Pregnant	Negative thought, fright
AgataBuo(2021)[22], Wright K(2020)[37]		increased stress and anxiety

## Conclusions

During these testing times of COVID-19 pandemic where each and every fragment of our society has undergone a significant traumatic issue in different areas of their social and psychological life. Due to the strict protocols issued by the governing bodies and the compliance and cognizance taken by the society helped to overcome and reduce the effects of this pandemic. However, many people have suffered a psychological setback of varying intensity. Large number of people experienced financial crisis, which has also contributed to their mental suffering. This has caused varying degrees of effect at all strata of society, including children as well. To overcome problems like these, implication of online counselling of the affected individual by a trained person for the issues pertaining to mental health, online teaching learning strategies for the students, work from home environment for most of the businesses and offices have proved to be helpful. Although the problem of this pandemic poses great social, psychological and financial implications, the quality and tendency of human nature always tries to overcome the difficult situations, and hopefully we all will come above this.
